# Non-Invasive Imaging of Phosphoinositide-3-Kinase-Catalytic-Subunit-Alpha (PIK3CA) Promoter Modulation in Small Animal Models

**DOI:** 10.1371/journal.pone.0055971

**Published:** 2013-02-05

**Authors:** Snehal M. Gaikwad, Lata Gunjal, Anitha R. Junutula, Arezoo Astanehe, Sanjiv Sam Gambhir, Pritha Ray

**Affiliations:** 1 Advanced Centre for Treatment, Research and Education in Cancer (ACTREC), Tata Memorial Centre, Navi Mumbai, Maharashtra, India; 2 Molecular Imaging Program at Stanford (MIPS), Departments of Radiology and Bioengineering, Department of Materials Science and Engineering, Bio-X Program, School of Medicine, Stanford University, Palo Alto, California; 3 Department of Obstetrics and Gynecology, University of British Columbia, Vancouver, British Columbia, Canada; University of California San Francisco, United States of America

## Abstract

Activation of the PI3K/Akt pathway, a critical step for survival in cancer cells is often associated with decreased sensitivity to several chemotherapeutic drugs. *PIK3CA* gene amplification is observed in 16–24% of epithelial ovarian cancer (EOC) patients in conjunction with p53 mutations. A 900 bp long *PIK3CA* promoter is shown to be negatively regulated by p53 in ovarian surface epithelial cells but the consequence of chemotherapeutic drug treatments on this promoter in ovarian cancer cells is largely unknown. We aim to study the modulation of this promoter by cisplatin using an improved fusion reporter in ovarian cancer cells and tumor xenografts by non-invasive imaging approach. A *PIK3CA* sensor was developed using a bi-fusion reporter from a newly constructed library of bi- and tri-fusion vectors comprising of two mutant far red fluorescent proteins (mcherry/mch and tdTomato/tdt), a mutant firefly luciferase (*fluc2*), and a PET reporter protein (*ttk*). *In vivo* imaging of mice implanted with 293T cells transiently expressing these bi- and tri-fusion reporters along with respective controls revealed comparable activity of each reporter in the fusion background and *fluc2-tdt* as the most sensitive one. Repression of the *PIK3CA* sensor by drugs was inversely proportional to cellular p53 level in a germline (PA1) and in an EOC (A2780) cell line but not in a p53 deficient EOC (SKOV3) cell line. Bioluminescence imaging of tumor xenografts stably expressing the *PIK3CA* sensor in PA1 and A2780 cells exhibited attenuating activity without any change in SKOV3 tumors expressing the *PIK3CA* sensor after cisplatin treatment. Sequential mutation at p53 binding sites showed gradual increase in promoter activity and decreased effects of the drugs. These newly developed *PIK3CA-fluc2-tdt* and the mutant reporter sensors thus would be extremely useful for screening new drugs and for functional assessment of *PIK3CA* expression from intact cells to living subjects.

## Introduction

The class 1 phosphatidylinositol-3-kinase (PI3K) family of lipid kinases phosphorylate the phosphatidylinositol 4,5 bisphosphate (PIP2) at the 3 position of the inositol ring that act as second cellular messenger for cell growth, survival, proliferation and morphology [1]. p110α, the catalytic subunit of the class I PI3K encoded by *PIK3CA* gene is de-regulated in many neoplasia by differential gene expression, amplification and mutation [2], [3], [4], [5]. In comparison to breast and hepatocellular carcinomas, amplification rather than mutation in *PIK3CA* is a common event in ovarian carcinomas and is frequently associated with *TP53* mutations [6], [7], [8]. About 16–24% of ovarian carcinomas harbour *PIK3CA* amplification irrespective of a histological subtype and is negatively associated with platinum sensitivity and PTEN over expression [7], [9]. While p110α mutations are extensively studied for targeted therapy with PI3K inhibitors, consequence of *PIK3CA* amplification for therapeutic intervention is yet to be fully investigated. Studies on ovarian cancer cell lines revealed that activation of the PI3K/AKT pathway may also lead to resistance to chemotherapy [10], [11].

Recent characterization of a 900bp long *PIK3CA* promoter fragment isolated from normal human ovarian surface epithelium (OSE) exhibited four p53 binding response elements and p53 mediated attenuation [12]. The same promoter isolated from Human Bacterial chromosome showed to bear NF-kβ, hypoxia inducible factor, heat shock protein and activator protein 1(AP1) binding sites [13]. Inhibition of nuclear translocation of NF-kβ or incubation with TNF-α resulted in down or up regulation of *PIK3CA* promoter activity [13]. Thus *PIK3CA* expression encounters complex regulation by several factors. However, the effect of the common therapeutic drugs (cisplatin and paclitaxel) on this *PIK3CA* promoter in ovarian cancer cells still remains to be investigated.

Non-invasive molecular imaging of living animals with reporter genes has opened up new avenues to understand fundamental molecular pathways in modern biomedicine [14], [15]. A variety of reporter genes have been developed for Optical, Magnetic Resonance and Radionuclide imaging techniques to study specific biological processes and monitor disease progression and therapy [16], [17], [18]. Modality specific reporter genes when used in combination add extra advantage of generating superior information with higher sensitivity, resolution and tomography. Multimodality imaging vectors generated by ‘fusion gene’ approach are most suitable for visualizing molecular events from both live cells and living organisms. Our previous multimodality fusion reporters (a combination of bioluminescent, fluorescent and PET reporters) [19], [20], [21], though accomplished significant achievements in non-invasive imaging of gene expression in living subjects [22], [23], [24] were limited for *in vivo* fluorescence imaging. The monomeric red fluorescent (mRFP1) protein used in these vectors is limited by lower quantum yield. The developments of fluorescent proteins as molecular tags have revolutionized the understanding of biological systems in live cells [25], [26], [27]. While the green fluorescent proteins and its mutants are suitable for imaging molecular events at cellular level, its red counterparts are optimal for small animal imaging. Some of these red fluorescent proteins (such as tdtomato, mTangarine, mStrawberry, mCherry etc.) have emission spectra near or slightly above 600 nm, a wavelength which experiences lesser attenuation and absorption in biological tissues. Further, constant molecular modification for functional improvement in bioluminescence reporters enhances the possibilities to construct improved and highly sensitive fusion reporters for non-invasive imaging.

To understand and monitor the *PIK3CA* promoter modulation by drugs, we generated a *PIK3CA* sensor with a newly constructed fusion reporter competent for both *in vitro* and *in vivo* imaging studies. Continuing improvement of our existing vectors [19], [21], [28] we first created several bi-fusion and tri-fusion reporters with higher *in vivo* optical imaging ability using two mutant red fluorescent proteins with better photon efficiency (tandem dimer Tomato or *tdt*) and or longer emission wavelength (mCherry or *mch*) and a codon optimized highly sensitive bioluminescence reporter (firefly luciferase2 or *fluc2*). Comparison of our newly constructed vectors with the existing ones carrying a mutated thermostable firefly luiciferase (*mtfl*), monomeric red flourescence protein (*mrfp1*) and truncated sr39thymidine kinase (*ttk*) showed the utility of these improved vectors for *in vivo* multimodality imaging as a proof of principle. We then evaluated the ability of *fluc2-tdt*, the most optimized vector to monitor the modulation of (*PIK3CA*) promoter in response to chemotherapeutic drugs in ovarian cancer cells and in tumor xenografts. Sequential mutations at the p53 binding sites gradually augmented the *PIK3CA* promoter activity and abolished the effects of drugs indicating a p53 mediated tight regulation of PIK3CA signalling in cellular homeostasis.

## Materials and Methods

### Chemicals

[8-^3^H] Pencyclovir was obtained from Moravek Biochemicals (Brea, CA). ^18^F-labeled 9-(4-[^18^F] Fluoro-3-hydroxymethylbutyl) guanine (FHBG) was synthesized at Stanford. The *tdtomato* and *mcherry* red fluorescent proteins were kind gift of Dr. R. Tsien (UCSD, CA). The *fluc2*, phRL–TK, Luciferase assay systems were purchased from Promega. D-luciferin and Coelenterazine were procured from Biosynth International (Switzerland). Cisplatin (cis-Diammineplatinum-(II)-dichloride), Paclitaxel, Adriamycin, β-actin antibody, secondary antibodies (i.e., anti-mouse and anti-rabbit) were obtained from Sigma while anti-p110α and anti-p53 antibodies purchased from Cell Signaling Technologies (Danvers, MA, USA).

### Construction of *mtfl*/*fluc2*-*tdt/mcherry-ttk* Fusion genes and *PIK3CA-fluc2-tdt* Vector

PCR amplification and standard cloning techniques were used to generate the *CMV-mtfl/fluc2*-*tdt/mch-ttk* tri-fusion and *CMV-mtfl/fluc2-tdt/mch bifusion* plasmids in pCDNA3.1(+) backbone using the existing triple fusion reporter vector CMV-*mfl-mrfp1-ttk* [28]. The *PIK3CA* promoter [12] was PCR amplified using primers (5′GTAAGATCTACTGCTCCTACGCTTTTC) and (3′ GCAGCTAGCTCGTGTAAACAAACAACG) and cloned in *CMV-fluc2-tdt* bifusion plasmid by replacing the CMV promoter. Positive clones were confirmed by PCR amplification, restriction digestion and sequencing.

### Site Directed Mutagenesis

*PIK3CA* promoter bearing four p53 binding sites was sequentially mutated using the mutagenic primers (Table 1) which consist of the substitutions at the core sequence of p53 binding sites by site directed mutagenesis. The colonies obtained were screened and verified by sequencing for desired mutations.

**Table 1:** The p53 binding sites in the PIK3CA promoter with core sequence (underlined) and the mutagenic primers.

p^53^ binding sites	Mutagenic primers
Site 1 CACCAAGACA	5′ GGTACGCAGCACTGTGACACTACCTTG 3′ F5′ CAAGGTAGTGTTACAGTGCTGCGTACC 3′ R
Site 2 TGGCATTACG CACCACGTCT	5′ CGCGAAAAATCCCCAGAATCTTCTGAATAG 3′ F 5′ CTATTCAGAAGATTCTGGGGATTTTTCGCG 3′ R
Site 3 ACGCTGGTTA CGGTTAGCCA	5′ TCCATAACCACGAGAATTAGCCACTGAC 3′ F5′ GTCAGTGGCTAATTCTCGTGGTTATGGA 3′ R
Site 4 AAGCAAGACG GCACATATTG	5′ TCGGGCGGAAAAGTGTGACGCAGGCG 3′ F5′ CGCCTGCGTTACACTTTTCCGCCCGA 3′ R

### Cell Lines, Transient Transfection, and Stable Cell Generation

293T (human embryonic kidney cells), A2780 (undifferentiated EOC cell) were obtained from ATCC (Manassas, VA, USA) and PA1 (germline ovarian cancer cells) was obtained from NCCS (Pune, India). The 293T cells were grown in MEM, while PA1 and A2780 cells were cultured in DMEM supplemented with 10% FBS and 1% penicillin/streptomycin solution [28]. The SKOV3 cells were cultured in McCoy’s medium supplemented with 10% FBS and 1% penicillin/streptomycin solution. All transient and stable transfections were carried out using the Superfect transfection reagent (Qiagen, Valencia, CA) and clonal selections were done by G418 selection. All the cell lines were tested for Mycoplasma contamination using EZdetectTM Hoechst Stain kit (#CCK008) (HiMedia Lab, Mumbai, India) and found to be negative. There is no dedicated cell line testing service provider in India.

### TK, RL, FL and β-GAL Activity

Thymidine kinase and β- Galactosidase enzyme activity assays were performed as previously described [28]. Renilla and Firefly luciferase assays were performed using Dual–Luciferase Reporter Assay System from Promega. Each of the luciferase reactions was measured in a Berthold luminometer for period of 1 sec. All transfection and drug treatment experiments were done in triplicates and repeated at least twice.

### Western Blotting

PA1 and A2780 cells stably expressing the *PIK3CA-fluc2-tdt* vector were treated with drugs for 2 and 24 hrs, lysed in passive lysis buffer. Equal amounts of protein from control and treated cells were resolved in SDS PAGE gel and probed with various primary and respective secondary antibodies.

### FACS Analysis and Immunofluorescence

293T cells transfected with various bi-fusiosn reporters for 24 hrs were trypsinized, washed in PBS and analyzed for tdTomato expression on the FL3 channel using a FACS Caliber (BD Biosciences, CA, USA). Analysis was performed using FlowJo Sofwatre (Tree Star).

To analyze the localization of p53, treated (cisplatin and paclitaxel) and control PA1 and A2780 cells were grown on cover slips, fixed in 4% paraformaldehyde, washed and blocked in 5% BSA followed by overnight incubation with primary antibody (1∶200). Cover slips were then washed, incubated with secondary antibody (2 hrs), counterstained briefly with DAPI and were observed under a Zeiss LSM 510 Meta confocal microscope. At least five representative fields were studied for p53 and DAPI staining.

### Fluorescence Imaging in Living Mice

Animal care and euthanasia were performed with the approval of the Administrative Panels on Laboratory Animal Care (A-PLAC) of Stanford University and Institutional Animal Ethics Committee (IAEC) of ACTREC. All images were acquired using a Maestro™ (Cambridge Research and Instrumentation, Woburn, MA) or Xenogen IVIS™ -200 (Xenogen Corp., Alameda, CA) optical imaging system. The mice were anesthetized, injected with 10×10^6^ cells transiently expressing the *fluc2-tdt-ttk* and *fluc2-tdt, mtfl-tdt-ttk, mtfl–tdt* fusion genes, and placed inside the imaging system [20]. For Maestro™ *in vivo* imaging system, the MSI data sets (cubes) were acquired with images spaced every 10 nm spectral interval in the 550 nm to 700 nm spectral range (Excitation filter 503–550 nm and Emission filter 580–700 nm). All the images were corrected for background auto-fluorescence. Region of interests were drawn on the site of interests and mean fluorescence intensity (MFI) were recorded. While using the Xenogen IVIS™ -200 system for imaging, the whole body image was acquired for 1 second with an excitation filter at 500–550 nm and an emission filter 575–600 nm. ROIs were drawn over implanted cell area and quantified by using Living IMAGE Software version 2.5. Fluorescence signal was recorded as maximum (photons/sec/cm^2^/sr).

### Bioluminescence Imaging in Living Mice

For *in vivo* bioluminescence imaging with Xenogen IVIS™-200 optical imaging system, mice implanted with 293T cells transfected with various single, bi and triple fusion reporter genes were anesthetized and placed in a light tight chamber and whole body images were obtained for 1 min after intra peritoneal injection of 100 µl D-luciferin (30 mg/ml) diluted in phosphate-buffered saline (pH 7) [20]. ROIs were drawn over the implanted cell area(s) and quantified by using the Living Image Software version 2.5. Bioluminescence signal was recorded as maximum (photons/sec/cm^2^/sr). For imaging with Berthold’s NightOwl II LB 983 optical imaging system, A2780 and PA1 cells stably expressing the *PIK3CA-fluc2-tdt* reporter were implanted in mice and imaged for bioluminescence when the tumors reached 5–8 mm size. The mice were then divided in two groups and one group was injected with cisplatin (8 mg/kg). ROIs were drawn over the tumors of control and cisplatin treated mice and quantified by using the Winlight Optimas Live Image Software 32. Bioluminescence signal was recorded as maximum (photons/sec/cm^2^) with a fixed angle at 2*pi.

### Small Animal PET Imaging in Living Mice

Mice were anesthetized, injected with 10×10^6^ 293T cells transiently expressing *fluc2-tdt-ttk, tdt, and fluc2* and *ttk* vectors. After fluorescence and bioluminescence imaging, mice were scanned using a microPET (P4, Simens) for 18F-FHBG uptake as described earlier [20]. Briefly, each mouse injected with 200 µCi of 18F-FHBG intravenously and scanned for 10 min after 1 hr of uptake. The microPET images were reconstructed with the ordered-subsets expectation maximization algorithm and analyzed using a Medical Imaging Data Examiner. Volumetric regions of interest were drawn over the tumors and the mean activities were recorded from the entire ROI. The percent injected dose (%ID/g) was calculated by dividing the ROI counts by the injected dose (decay corrected).

## Results

### Multimodality Imaging of a Newly Developed Optimized Triple Fusion (fluc2-tdt-ttk) Reporter in Living Mice

We aim to construct new triple and bi-fusion vectors by modifying the existing fusion reporters (CMV-*mtfl-mrfp1-ttk*) to achieve high enough sensitivity, useful for imaging different molecular pathways *in vivo*. To improve the lower fluorescence quantum yield, extinction coefficient and photostability of mRFP1, Shaner *et al* (2008) [29] performed a rigorous mutagenesis screen focusing on selected amino acids. Of all those mutants, we chose to work with *tdtomato* and *mCherry* protein due to their higher quantum yield, brightness, and longer emission wavelength. To improve the sensitivity of the bioluminescence component of the fusion vectors, a mutant of firefly luciferase (*fluc2*) having 5–10 fold higher light output than original *fluc* was used. Amongst all the newly constructed triple fusion reporters, *fluc2-tdt-ttk* showed highest fluorescence and bioluminescence but moderate TK activity as measured by FACS, FL and TK assays from 293T cells transiently expressing the new generation triple fusion vectors with appropriate controls (data not shown).

**Figure 1:**
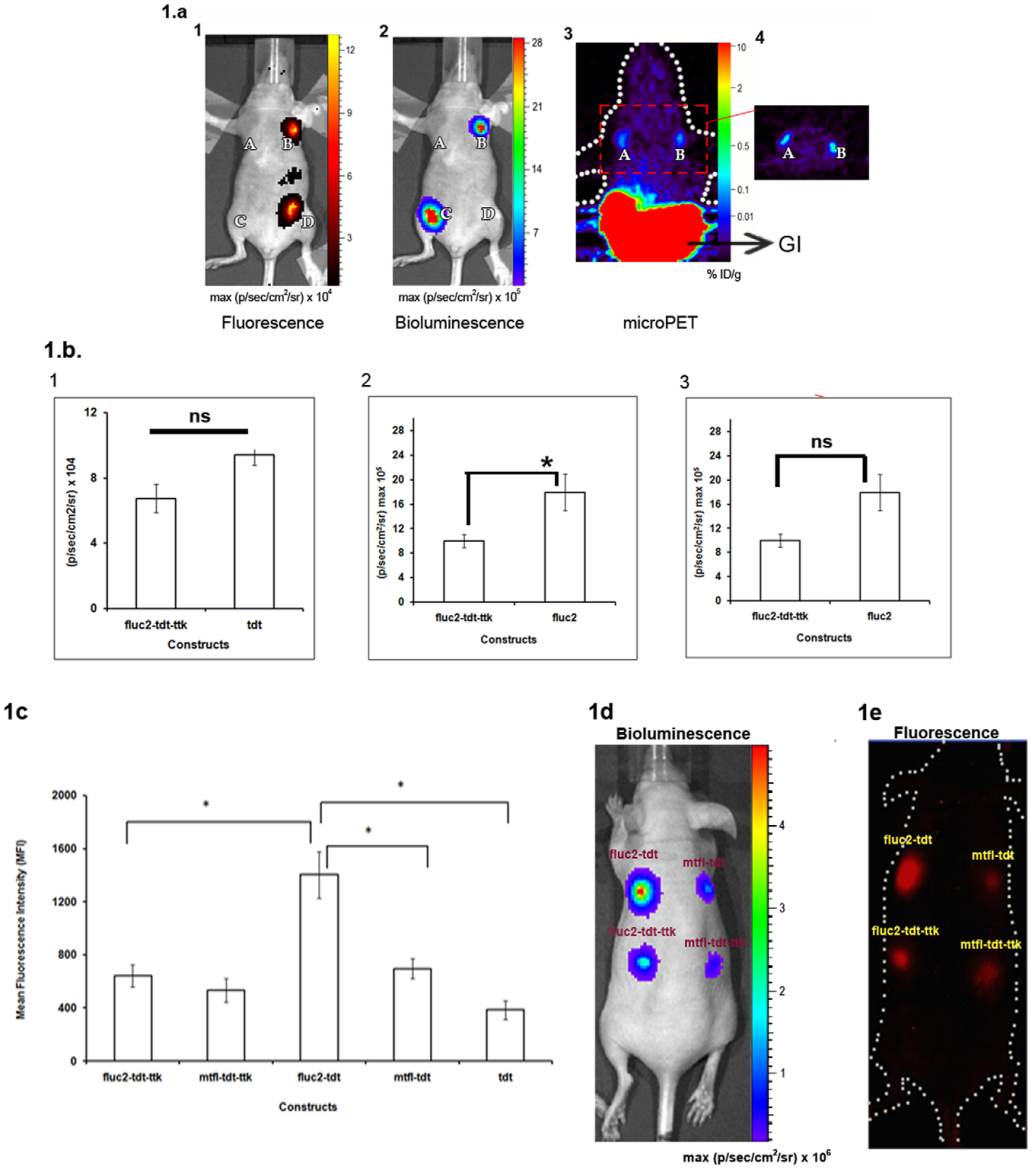
Multimodality imaging of the new generation bi- and tri-fusion vectors in living mice. 1a1 and 1a2. Fluorescence and Bioluminescence imaging. 10×10^6^ of 293T cells transfected with *CMV-ttk (A), CMV-fluc2-tdt-ttk (B), CMV-fluc2(C),* and *CMV-tdt (D)* plasmids were implanted subcutaneously in living mice (n = 3) and were imaged first for fluorescence and then for bioluminescence after injection of D- luciferin using IVIS imaging system. Signals were seen only from the cells expressing *CMV-tdt* and *CMV-fluc2-tdt-ttk* vector (for fluorescence) and from cells expressing *CMV- fluc2* and *CMV-fluc2-tdt-ttk* reporter for bioluminescence. Signals were recorded as max (pixel/sec/cm^2^/steradian). 1a3. microPET imaging. Mice described in 1a1 were injected with 200 µCi of 18F-FHBG and microPET imaging was performed after 1 hr for 10 minutes. Specific uptake of 18F-FHBG was seen in cells expressing the *CMV-ttk* and *CMV-fluc2-tdt-ttk* vectors. High nonspecific accumulation of 18F-FHBG was seen in the gastro-intestinal tract (GI). 1a4. Coronal section of the same microPET image described in 1a3. 1b. Graphical representation of the quantified fluorescence (1b1), bioluminescence (1b2) and microPET (1b3) signals. 1b1 and 1b2. Fluorescence (B and D) and bioluminescence (B and C) signals were calculated for the respective ROIs drawn over the sites of implanted cells. The SEM represents 3 experiments (ns.- statistically non-significant and *indicates p<0.05). 1b3. Percent injected dose (%ID/G) of 18F-FHBG uptakes were calculated for the respective ROIs drawn over the implanted cell (A and B) which showed similar uptake (ns). 1c. Comparative analysis of the fluorescence activity of the new bi and trifusion vectors in cell culture: 293T cells were transiently transfected with *CMV-fluc2-tdt-ttk*, *CMV-mtfl-tdt-ttk, CMV-mtfl-tdt* and *CMV-fluc2-tdt* plasmids and FACS analysis was done from equal number of cells after 24 hrs. All the experiments were performed in triplicate (*indicates p<0.05). 1d & 1e. Fluorescence and bioluminescence imaging of the new bi and triple fusion vector. 10×10^6^ of 293T cells transfected with *CMV-fluc2-tdt-ttk*, *CMV-mtfl-tdt-ttk, CMV-mtfl-tdt* and *CMV-fluc2-tdt* plasmids were implanted on the dorsal side of a nude mouse and imaged for fluorescence (1d) using Maestro system and bioluminescence (1e) as described above. Cells expressing *CMV-fluc2-tdt* clearly exhibited highest fluorescence and bioluminescence signals among all group of cells.

To test the efficiency of our most sensitive vector *fluc2-tdt-ttk in vivo,* we implanted 5×10^6^ 293T cells transiently expressing *fluc2-tdt-ttk* (site A), *fluc2* (site C), *tdt* (site D) and *ttk* (site B) reporter genes at four different sites in nude mice (n = 3) and imaged by fluorescence, bioluminescence and microPET scanner after 24 hrs. The *in vivo* fluorescence and bioluminescence imaging noticeably showed very high levels of fluorescence and bioluminescence signals from cells expressing *fluc2-tdt-ttk* compared to cells expressing *tdt* or *fluc2* reporters (Figure 1a1 & 1a2). microPET imaging with 18F-FHBG showed low but distinct accumulation of the tracer at the implanted sites of cells expressing the triple fusion reporter and *ttk* reporter alone (Figure 1a3 & 1a4). A graphical analysis of the quantified signals of all the modalities showed that the fluorescence, bioluminescence and microPET signals of triple fusions are lower than the signals of *tdt* or *fluc2* or *ttk* vector alone which met significance only for bioluminescence signal (Figure 1b1, 1b2 & 1b3).

### The Fluorescence and Bioluminescence Imaging of the Most Sensitive Fusion Reporter (fluc2-tdt)

We next sought to compare the reporter activities of bi (*fluc2-tdt* & *mtfl-tdt*) and triple fusions (*fluc2-tdt-ttk* & *mtfl-tdt-ttk*) by FACS and *in vivo* fluorescence and bioluminescence imaging. When comparisons were made between the triple fusion and bi-fusion vectors in transiently transfected 293T cells, *fluc2-tdt* exhibited very high fluorescence activity (even higher than cells expressing *tdt* alone) (Figure 1c). We tried to compare different cell numbers (10,000 to 5 million) transiently transfected with all the four vectors and implanted in mice. As shown in Figure 1d & 1e, very bright fluorescence and bioluminescence signals were visible from the site of cells (50,000) expressing the *fluc2-tdt* fusion reporter. The expression of *fluc2-tdt* could also be visible from much lower number of cells (10,000) (data not shown).

### Monitoring Drug Induced Modulation of PIK3CA Promoter with a Unique Sensor (PIK3CA-fluc2-tdt)

Astanehe *et al* (2009) [12] demonstrated that binding of p53 suppresses the *PIK3CA* promoter activity in normal OSE. Both cisplatin and paclitaxel, the standard chemotherapeutic drugs for ovarian cancer are known to induce p53 mediated cell death [30], [31]. To study the modulation of *PIK3CA* promoter by chemotherapy drugs in ovarian cancer cells, we cloned the *fluc2-tdt*, under the *PIK3CA* promoter [12]. A dose dependent treatment of cisplatin and paclitaxel for 2 hrs indicated that a 5 µg/ml concentration of both drugs were able to decrease the promoter activity in PA1 cells (data not shown). Treatments with three drugs [cisplatin, paclitaxel and adriamycin (1 µM/ml)], significantly decreased the *PIK3CA* promoter activity (Figure S1a) but not the co-transfected TK promoter activity (*pTK-hrl*) (Figure S1b) in PA1 cells. Similar results were observed in A2780 cells transiently transfected with *PIK3CA-fluc2-tdt & pTK-rluc* and treated with cisplatin, paclitaxel & adriamycin (data not shown).

### Cisplatin and Paclitaxel Treatments in PA1 and A2780 Cells Stably Expressing PIK3CA-Fluc2-tdt Exhibit PIK3CA Promoter Modulation and p53 Activation

**Figure 2:**
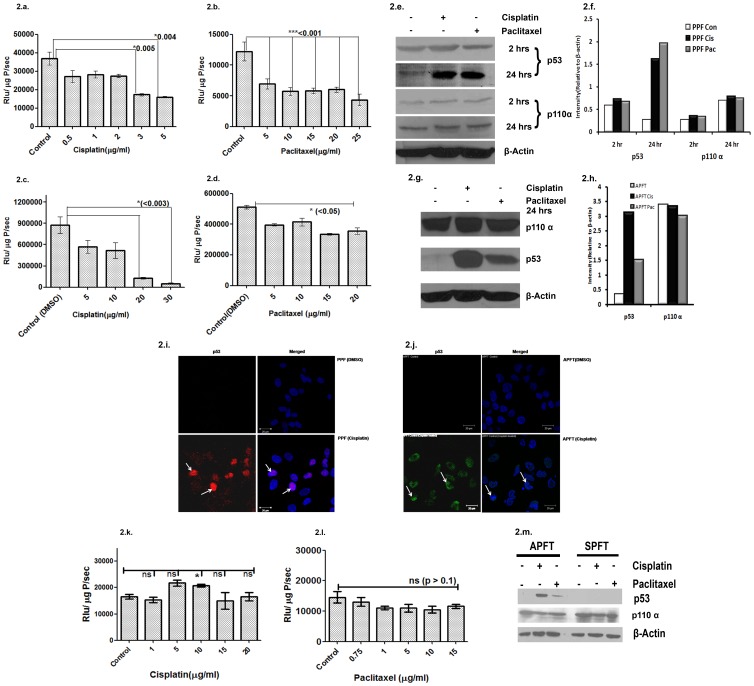
In vitro studies on effect of Cisplatin and paclitaxel on PIK3CA promoter and p53. 2a-d and 2k -2l. *PIK3CA* promoter activity in drug treated PA1-*PIK3CA-fluc2-tdt* (PPF), A2780-*PIK3CA-fluc2-tdt* (APFT) and SKOV3-*PIK3CA-fluc2-tdt* (SPFT) (2k and l) cells. A dose dependent decrease in luciferase activity (*PIK3CA* promoter activity) was observed on increasing concentrations of cisplatin treatments in PPF cells (at 3 & 5 µg/ml cisplatin, p<0.03) (2a) and in APFT cells at 20 and 30 µg/ml cisplatin (p<0.003) (2c). Similar dose dependent decrease in luciferase activity was obtained on increasing concentration of paclitaxel treatment in PPF up to 25 µg/ml, p<0.001) (2b) and in APFT cells upto 20 µg/ml paclitaxel, p<0.05 (2d) No such dose dependent change was observed on treatments with increasing concentration of cisplatin and paclitaxel (2k and 2l) in SPFT cells. 2e-2 h. Endogenous expression of p110α and p53 proteins after drug treatments. Western blot analysis of p53 protein from lysates of cisplatin and paclitaxel treated PPF and APFT cells showed induction in p53 level after 24hrs; however no change was observed after 2hrs. The p110α levels did not show any change after drug treatment (2e & 2g). The densitometric analysis representing the same is shown in 2d & 2h. The p53 mutant status in SPFT cells was verified by western blotting (2 m). 2i & 2j. Nuclear localization of p53 after treatment in cisplatin treated cells. p53 protein (red in 3g and green in 3h) showed nuclear localization upon treatment with cisplatin for 2hrs as compared to the vehicle treatment in PPF and APFT cells. DAPI (blue) indicated the nuclear staining.

Treatments with cisplatin and paclitaxel showed attenuated luciferase activity in PA1-*PIK3CA-fluc2-tdt* (PPF) and A2780-*PIK3CA-fluc2-tdt* (APFT) cells following the same trend observed in transient expression study (Figure 2a–2d). Interestingly same concentration of drug (5 µg/ml) (either cisplatin or Paclitaxel) exerted different levels of promoter attenuation to A2780 (less sensitive) and PA1 (highly sensitive) cells. Western blot analysis of the same lysates did not show any change in the endogenous p110α level but the p53 level significantly increased after 24 hrs of drug treatments as compared to 2 hrs (Figure 2e, 2f & 2g, 2 h). In APFT cells, the level of p53 activation by cisplatin seemed to be higher than the level induced by paclitaxel (8.1 fold vs. 4.4 fold) (Figure 2 h). Immunofluorescence study clearly demonstrated nuclear localisation of p53 protein in cisplatin treated PPF and APFT cells (Figure 2i & 2j).

To investigate the effect of chemotherapeutic drugs on *PIK3CA* promoter in absence of endogenous p53 protein, stable clones of SKOV3 (a p53 deficient cell line) cells expressing the *PIK3CA* sensor was developed (SPFT). The mutant background was verified by western blotting (Figure 2 m). The *PIK3CA* promoter activity in these SPFT cells did not show any attenuation when treated with increasing concentrations of cisplatin and paclitaxel for 2 hrs (Figure 2k & 2l).

### Imaging of Cisplatin Induced Modulation in PIK3CA Promoter Activity in Tumors of Living Mice

**Figure 3:**
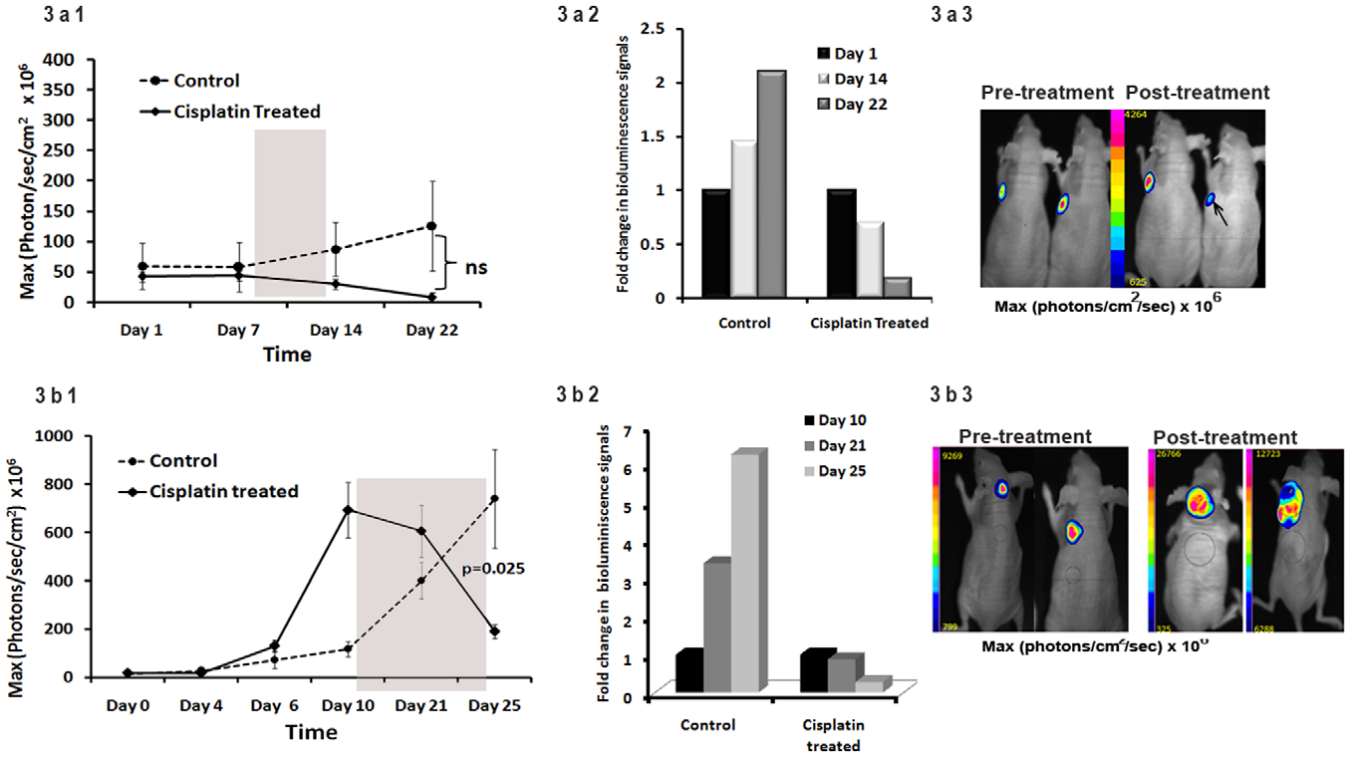
Non-invasive imaging of PIK3CA promoter modulation in tumor xenografts of living mice after cisplatin treatment. 3a. Bioluminescence signal of PIK3CA promoter in PPF tumors. 3a1. Graphical representation of the kinetics of *PIK3CA* promoter modulation. The luciferase activity in the control group of mice (n = 3) increased with time while that of the treated group (n = 3) attenuated from the fourteenth day of treatment till the 22^nd^ day (end point) after one treatment (8 mg/kg) (shaded area represented the days of treatment). At 22^nd^ day, measurable attenuation in the bioluminescence signal between the control and treated mice was evident however it did not reach statistical significance. (Day 1 represents the day prior to treatment). 3a2. Graphical representation of fold-changes in the bioluminescence signals. The temporal fold change in bioluminescence signal (post treatment signal/pre treatment signal) demonstrated augmented bioluminescence in the control group and attenuation in the treated group. 3a3. Representative bioluminescent images of the mice bearing PPF tumors. Mouse from the control and treated group exhibited specific and similar intensity signals which decreased after treatment (arrowhead). 3b. Bioluminescence signal of PIK3CA promoter in APFT tumors after cisplatin treatment. 3b1. Graphical representation of the kinetics of *PIK3CA* promoter modulation. The bioluminescence signal in the control mice (n = 3) increased with time while that of the treated group (n = 4) showed a slight decrease at 11th day after first treatment and significant attenuation at 15^th^ day after second treatment (p<0.025). 3b2. Graphical representation of fold-changes in the bioluminescence signals. The temporal fold change in bioluminescence signals (post treatment signal/pre treatment signal) demonstrated augmented bioluminescence in the control group and but attenuation in the treated (Day 10 represented signal prior to treatment). 3b3. Representative bioluminescent images of the mice bearing APFT tumors. Mouse from control and treated group exhibited specific signals which decreased only in treated mouse as shown by an arrow.

Non-invasive optical imaging is a great approach to track molecular events in living animals and correlate the findings with *in vitro* results. To explore the kinetics of *PIK3CA* promoter modulation by cisplatin *in vivo*, we used two different tumor xenograft models (PA1-*PIK3CA-fluc2-tdt* and A2780-*PIK3CA-fluc2-tdt*) with differential growth pattern. For each model, six million cells were subcutaneously implanted in nude mice and tumor growth was monitored by bioluminescence imaging (Figure 3a & 3b). Once the tumors were palpable, either one or two cycles of cisplatin (8 mg/kg/week) [32] was injected intraperitoneally in three nude mice for three days a week. To avoid toxicity, we chose to divide each dose in three parts. Since ovarian germline tumors are more sensitive to cisplatin in comparison to epithelial ovarian tumors and PA1 cells in our study showed higher promoter attenuation mediated by cisplatin (Figure 2a & 2e), we decided to treat the PA1 tumor bearing mice (n = 6) with one cycle and A2780 tumor bearing mice (n = 7) with two cycles of cisplatin. Attenuation in luciferase activities for PA1 model (4.4×10^8^±2.2×10^8^ p/sec/cm^2^ to 3×10^8^±1.9×10^8^ p/sec/cm^2^) (∼0.7 fold) were detected at 14^th^ day of completion of treatment which further decreased (0.82×10^8^±5.7×10^7^ p/sec/cm^2^) (∼0.2 fold) with time (22^nd^ day). The control mice, however, had increased luminescence and tumor growth with time (5.8×10^8^±4.1×10^8^ p/sec/cm^2^ to 8.7×10^8^±4.5×10^8^ p/sec/cm^2^ to 1.3×10^9^±7.4×10^8^ p/sec/cm^2^) (Figure 3a1 & 3a2). In the A2780 tumor model (n = 4), which exhibited a faster growth kinetics, the bioluminescence signal did not decrease after first treatment (6.93×10^9^±1×10^9^ p/sec/cm^2^ to 6.1×10^9^±1×10^9^p/sec/cm^2^) (0.9 fold) at 11 days but decreased rapidly (1.9×10^9^±3×10^8^ p/sec/cm^2^) (∼0.27 fold) after the completion of two treatments at 15 days. The control mice (n = 3) showed increased bioluminescence and tumor growth (1.2×10^9^±3×10^8^ p/sec/cm^2^ to 4×10^9^±7.6×10^8^ p/sec/cm^2^ to 7.4×10^9^±2×10^9^ p/sec/cm^2^) over time (Figure 3b1 & 3b2). The bioluminescence signals in the cisplatin treated mice at day 15 post-treatment showed a significant decrease (p = 0.025) as compared to the control mice (Figure 3b1).

**Figure 4:**
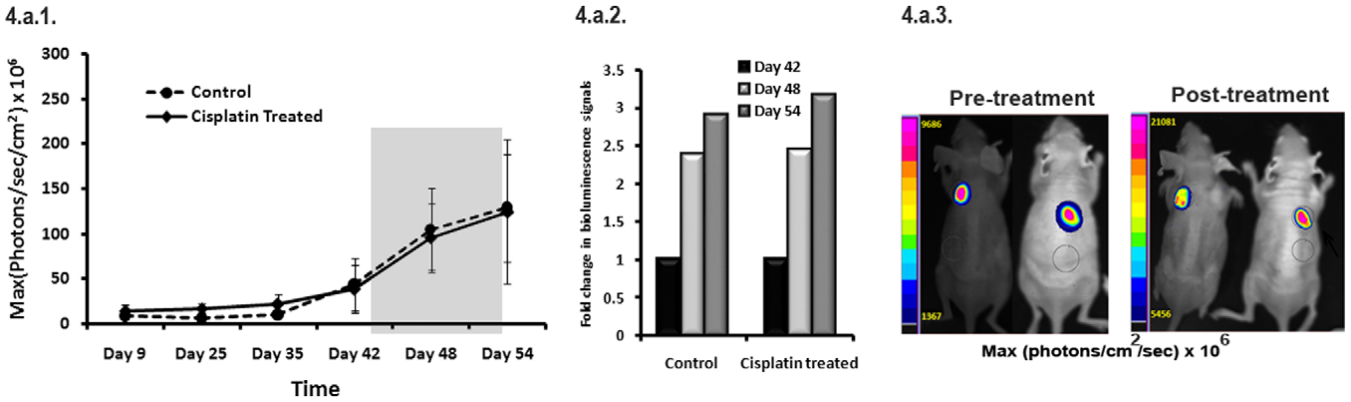
PIK3CA promoter activity in SKOV3 (p53 deficient) tumor xenograft model after cisplatin treatment. 4a1. Graphical representation of the kinetics of *PIK3CA* promoter modulation in the control and cisplatin treated mice. The bioluminescence signal in both control and treated mice increased with time even after two rounds of cisplatin treatment (n = 5). 4a2. Graphical representation of fold-changes in the bioluminescence signals of the control and treated mice. The temporal fold change in bioluminescence signals calculated ratiometrically (post treatment signal/pre treatment signal) demonstrated augmented bioluminescence in both the control and treated group (Day 42 of 4c1 is represents Day1 i.e. signal prior to treatment). 4a3. Representative bioluminescent images of the mice bearing tumors of SPFT cells before and after treatment. Mouse from control and treated group exhibited specific [Fig. 4a3 (pre-treatment)] signals which did not decrease even after two treatments of cisplatin [Fig. 4a3 (post-treatment)].

In contrast to the PPF and APFT tumor models, the bioluminescence signals of SKOV3 tumor xenografts (n = 5) did not decrease rather exhibited an increase after first treatment (3.9×10^7^±2.7×10^7^ p/sec/cm^2^ to 9.6×10^7^±3.8×10^7^ sec/cm^2^) (2.4 fold) of cisplatin at 7^th^ day, which further increased to 1.2×10^8^±8×10^7^ p/sec/cm^2^ (3.1 fold) after completion of two treatments at 24 days. The control mice (n = 5) also showed increased bioluminescence and tumor growth (4.4×10^7^±2.9×10^7^ p/sec/cm^2^ to 1.1×10^8^±4.5×10^7^ p/sec/cm^2^ at day 7 to 1.3×10^8^±5.9×10^7^ p/sec/cm^2^) (∼3 fold) over time (Figure 4a1 & 4a2). The representative images of the bioluminescence signals of pre- and post- treatment in PA1, A2780 and SKOV3 tumor models are shown in the Figure 3a3, 3b3 and 4a3.

### Mutations at p53 Binding Sites Augment *PIK3CA* Promoter Activity

**Figure 5:**
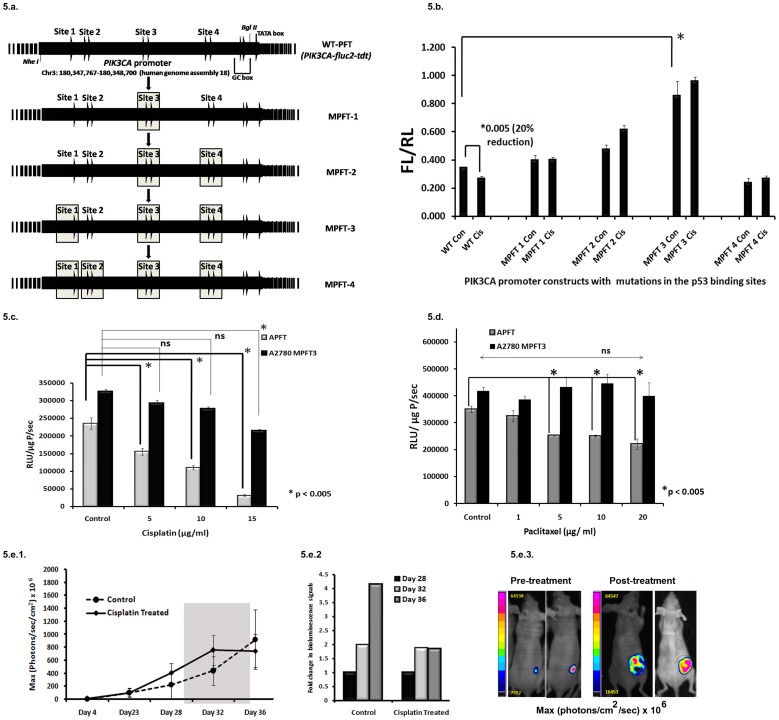
Mutations at p53 binding sites augment PIK3CA promoter activity. 5a. Substitutions in core sequence of p53 binding sites of *PIK3CA* promoter by sequential site directed mutagenesis to obtain four mutant *PIK3CA-fluc2-tdt* reporters (MPFT-1, MPFT-2, MPFT-3 and MPFT-4). 5b. Sequential mutation augments *PIK3CA* promoter activity. Ratiometric analysis of wild type and mutant *PIK3CA-fluc2-tdt* reporters with *pTK-hRL* from transiently transfected A2780 cells with and without cisplatin treatment (2 hrs) were graphically represented. Twenty percent reduction in WT-PFT activity (p<0.005) without any attenuation in the mutant promoters (MPFT-1, MPFT-2 and MPFT-3) were observed post cisplatin treatment. These mutant promoters showed a gradual augment of PIK3CA expression compared to WT-PFT with MPFT 3 showing a maximum of 2.5-fold increase (*Fig. 5b). The MPFT-4 promoter showed an overall decrease in *PIK3CA* expression in comparison to WT-PFT. The SEM represents triplicate experiments. 5c and 5d. Effect of cisplatin and paclitaxel in A2780-MPFT3 cells. Treatment with increasing concentrations of cisplatin (5 and 10 µg/ml) and paclitaxel (1–20 µg/ml) exhibited attenuated luciferase activity in APFT cells but not in A2780-MPFT3 cells except at very high dose of cisplatin (15 µg/ml). The SEM represents triplicate experiments. 5e. Non invasive imaging of cisplatin treated A2780-MPFT3 tumor xenografts. 5e1. Graphical representation of the kinetics of *PIK3CA* promoter modulation. The bioluminescence signal in both control and treated mice increased with time even after two rounds of cisplatin treatment (n = 3). 5e2. Graphical representation of fold-changes in the bioluminescence signals. The temporal fold change in bioluminescence signals calculated ratiometrically (post treatment signal/pre treatment signal) demonstrated augmented bioluminescence in both the control and treated group (Day 28 of 5e1 is represents Day1 i.e. signal prior to treatment). 5e3. Representative bioluminescent images of the mice bearing tumors of A2780-MPFT3 cells. Mouse from control and treated group exhibited specific [Fig. 5e3 (pre-treatment)] signals which did not decrease even after two treatments of cisplatin [Fig. 5e3 (post-treatment)].

Mutation in one of the four p53 binding sites (site 4) in PIK3CA promoter showed 50% less attenuation in presence of conditionally activated p53 protein [12]. We performed a series of site directed mutagenesis to sequentially abolish the binding of p53 in the promoter (Figure 5a) and measured luciferase activities from transiently transfected A2780 cells with wild and the four mutant *PIK3CA*-*fluc2-tdt* reporters along with *pTK-hrl* gene after cisplatin treatment. Mutation at site 3 alone (MPFT-1), sites 3&4 (MPFT-2), sites 3,4&2 (MPFT-3) and sites 3,4,2&1 (MPFT-4) did not exhibit any signal attenuation by cisplatin in comparison to the 20% reduction showed by the wild type promoter (Figure 5b). Intriguingly, three of these mutant promoters (MPFT-1, 2, 3) showed gradual augmentation of *PIK3CA* expression in comparison to the wild type promoter with MPFT-3 showing the maximal increase (2.5-fold). The MPFT-4 promoter showed overall decrease in *PIK3CA* expression in comparison to the wild type and other mutants. This attenuated activity of MPFT-4 was unexpected however after careful analysis of the *PIK3CA* promoter two important transcription factor binding (NF-kβ & HIF-1 ancillary sequence) sites were found to overlap at site 2 (Table 2). Our preliminary result suggested that TNF-α induced up regulation of wild type *PIK3CA* promoter was lost in MPFT-4 construct (data not shown) and further experiments are ongoing. Finally to assess the modulation of mutant *PIK3CA* promoter *in vivo*, stable clone of A2780 cells expressing the MPFT-3 construct was generated and treated with cisplatin and paclitaxel. In corroboration with the transient transfection result, the stable clones (APFT vs. A2780-MPFT3) showed an increase in luciferase activity (1.4 fold). However, no significant change in the promoter activity of A2780-MPFT3 cells was observed with increasing concentration of cisplatin and paclitaxel (Figure 5c & 5d) except for the high concentration (15 µg/ml) of cisplatin. The APFT cells did exhibit attenuated promoter activity with all concentrations of cisplatin and paclitaxel. Further to observe the effect of cisplatin on MPFT-3 promoter *in vivo*, six million A2780-MPFT3 cells were implanted and tumors were allowed to grow in nude mice (n = 3). As expected, the bioluminescence signals in treated mice did not decrease rather exhibited an increase after first treatment (4×10^8^±1.4×10^8^ p/sec/cm^2^ to 7.5×10^8^±2.3×10^8^ sec/cm^2^) (2.4 fold) at 7^th^ day, which remained constant to 7.5×10^8^±2.5×10^8^ p/sec/cm^2^ after completion of two treatments at 24 days. The control mice (n = 3) also showed increased bioluminescence and tumor growth (2.3×10^8^±2×10^8^ p/sec/cm^2^ to 4.4×10^8^±2.2×10^8^ p/sec/cm^2^ at day 7 to 9.2×10^8^±4.5×10^8^ p/sec/cm^2^) (∼4 fold) over time (Figure 5e1 & 5e2). The representative images of the bioluminescence signal in mice pre- and post- treatment in A2780-MPFT3 tumor models are shown in the Figure 5e3.

**Table 2:** Transcription factor binding sites in PIK3CA promoter*.

Sequence	Transcription Factors	Position (bp)
TTACAACAAAAGACCAGTAGGGGGA	SOX/SRY-sex/testis determining and related HMG box factors	24–48
CACCAAGACA	Half p53 binding element (**site 1**)	142–151
TGGCATTACG CCCCACGTCT	p53 binding site **(Site 2)**	201–210 220–229
CGCGAAAAATCCCCC	NF kβ (overlap at **site 2**)	209–223
AAGTGAGTCAAAG	AP1	320–332
ACGCTGGTTA CGGTTAGCCA	p53 binding site (**Site 3**)	457–466481–490
TAGAAACAAATATACTA	Fork head domain factors	608–624
CACGTACGCTGT	HIF-1	624–636
GATGACACAACA	AP1	642–653
AAGCAAGACG GCACATATTG	p53 binding site (**Site 4**)	732–741753–762

Note: *As analysed by the Genomatix software and using references from Astanehe et al, 2008 and Yang et al, 2009.

## Discussion

The standard therapeutic regimen for treating ovarian cancer is a combinatorial treatment of platinum (cisplatin or carboplatin) and taxol (paclitaxel) based drugs often followed by de-bulking surgery [33]. Both these drugs induce apoptotic pathways either by forming DNA adducts or by inducing cell cycle arrest and subsequent cell death [30]. Activation of p53 is a central molecular event that guides the cells to follow either a survival or an apoptotic route after the genotoxic insult. Activation of p53 might down-regulate the PIK3CA/Akt signalling as indirectly evidenced by association of *PIK3CA* gene amplification with p53 mutations in ovarian carcinoma [7], [9].

p110α, the catalytic subunit of class I PI3K and encoded by *PIK3CA* gene is tightly regulated in normal cells. PI3KCA activation either by mutation or gene amplification initiates a signal transduction pathway that promotes growth, metabolism, and survival in cancer cells [5], [8], [9]. The putative ∼900 bp *PIK3CA* promoter carries several important binding sites for p53, NF-κβ, HIF, and AP1 transcription factors [12], [13]. While direct binding of p53 attenuates the *PIK3CA* promoter activity, inhibition of NF-kβ degradation or treatment with TNF-α results in moderate induction. However, none of these studies have attempted to find the effect of a chemotherapeutic drug on this promoter.

Non-invasive imaging of molecular events in small animals has become a standard practice to evaluate new drugs. Reporter genes or combination of reporter genes which can be used with multiple imaging devices add the advantage of collecting multiple signals from deep inside the body, with higher sensitivity and specificity over reporter genes suitable for only a single imaging modality [20], [34]. Thus fusion reporter genes have gained popularity in preclinical imaging. Over the years we have built a small library of fusion reporter vectors and have been applying to monitor tumor metastasis, cell/stem cell trafficking, stem cell therapy, and other areas [22], [23], [24]. These fusion vectors comprise of a bioluminescent (either *fluc* or *hrluc* and their mutants), a fluorescent (either *gfp* or red fluorescent proteins and their mutants) and a PET reporter (*sr39* mutant thymidine kinase or wild type thymidine kinase) gene joined by small peptide linkers [20], [21]. Our previous fusion reporters using a monomeric red fluorescent protein1 emitting light at 608 nm wavelength have suffered at sensitivity due to poor quantum yield and low photostability of the protein. Recently Tsien’s group at UCSD developed several mutant red fluorescent proteins of which tdTomato is the most optimal for *in vivo* imaging. Even a low number of breast cancer cells expressing tdTomato can be imaged noninvasively from living mice including metastasis to lymph nodes [35]. The mCherry protein though having lower quantum yield has excitation spectra at 613 nm, a far red region optimal for *in vivo* imaging. When both these RFP mutants (tdTomato and mCherry) were tried as triple fusion partners, only tdTomato fusions could retain significantly higher fluorescence activity [29]. Among the bi-fusions and triple fusions carrying tdTomato, the bi-fusions are the brightest.

In parallel to improvement of fluorescent proteins, the luciferase genes were also attempted for enhancement of light output by mutagenesis, deletion of cryptic transcription factor binding sites and codon optimization for improved mammalian expression. The optimized version of *fluc (fluc2)* from Promega is able to generate 10-fold higher signals than *fluc* gene and this *fluc2* was used to generate the third generation fusion reporters by replacing the mutated thermostable *fluc* (*mtfl*). Interestingly, the newly developed *fluc2* containing fusion reporter (*fluc2-tdt*) show higher fluorescence along with higher luciferase activity than the previous fusions. This apparent increase in overall fluorescent activity after introduction of *fluc2* protein maybe due to the change in the quaternary structure resulting in better exposure of the flurophores present in the fluorescent proteins.

To understand the regulation of *PIK3CA* promoter in ovarian cancer cells, we utilized this optimized *fluc2-tdt* fusion reporter to monitor the effects of cisplatin and paclitaxel from intact cells to living animals. To our best of knowledge this is the first report of understanding the kinetics of drug induced *PIK3CA* promoter modulation. Our newly constructed *PIK3CA (PIK3CA-fluc2-tdt)* sensor exhibited significant attenuation after treatment with three chemotherapeutic drugs (cisplatin, paclitaxel and adriamycin) commonly used for ovarian cancer patients in two different cancer cells. The similar treatments did not affect the *TK* (Thymidine kinase) promoter in PA1 and A2780 cells. The level of attenuation varied between the cell lines with PA1 cells being more sensitive (2.3 fold reduction in promoter activity) than A2780 cells (1.3 fold) at same concentrations of cisplatin or paclitaxel (5 µg/ml) reflecting their respective clinical behaviour. All these drugs are known to induce cell death directly or indirectly via p53 mediated apoptotic pathways [31], [32], [36]. Reduction in promoter activity but no detectable variation in the endogenous p110α level after cisplatin or paclitaxel treatments indicates the strength of *fluc2-tdt* fusion reporter and reporter assay technique in measuring subtle changes of *PIK3CA* at molecular level. The endogenous p53 protein level, however, was significantly induced by the drugs. Activation of p53 by these drugs thereby leads to increased binding of p53 to the *PIK3CA* promoter and its suppression. This drug mediated attenuation of *PIK3CA* activity due to increased binding of activated p53 was not detected in a p53 deficient EOC (SKOV3) cell line. Both cisplatin and paclitaxel with increasing concentrations were not able to attenuate the *PIK3CA* promoter in these cells. The *in vivo* imaging kinetics showed a decrease in bioluminescence signal in both PA1 and A2780 tumors (expressing *PIK3CA-fluc2-tdt*) after cisplatin treatment. While a single treatment of cisplatin caused measurable reduction in luminescence activity in PA1 tumors at 14^th^ day, it did not effectively reduce the PIK3CA promoter activity in A2780 tumors. Two successive treatments were required to achieve significant reduction in luminescence activity in A2780 tumors. This differential effect of cisplatin on two different tumor types correlates well with their origin as ovarian germline tumors are known to be more sensitive to cisplatin in comparison to epithelial ovarian tumors. In corroboration with the *in vitro* results, cisplatin treatment *in vivo* also did not reduce the bioluminescence signals of SKOV3 tumor xenografts stably expressing the PIK3CA sensor indicating that presence of p53 protein is essential for *PIK3CA* regulation in ovarian cancer.

Surprisingly, sequential deletion of the p53 binding sites exhibited a gradual increase in the normal promoter activity indicating a temporary relief of p53 mediated suppression. Cisplatin induced attenuation of these mutant promoters were abolished. Inability to down regulate the mutant *PIK3CA* promoter by cisplatin was also reflected through non-invasive imaging of tumor xenografts stably expressing MPFT3, the promoter carrying three mutated p53 binding sites. Surprisingly, MPFT4 carrying mutations at all the four p53 binding sites showed an overall attenuation which might occur due to destabilization of co-operative bindings of other transcription factors required for PIK3CA expression. Indeed after careful analysis of *PIK3CA* promoter, site 2 was found to contain overlapping binding sites for NF-kβ and Hypoxia Inducible Factor ancillary sequence (Table 2). We are currently analyzing the role of these two factors in regulation of this MPFT-4 promoter. The PIK3CA pathway is one of the most crucial cellular defence mechanisms and therefore requires tight regulation at transcriptional and translational level. These newly developed *PIK3CA-fluc2-tdt* and the mutant reporter sensors could act as screening tools for potential new drugs. The power of these vectors from translating such information from single cells to the organism level will facilitate wide application in functional assessment of PIK3CA pathway for future therapeutic evaluation.

## Supporting Information

Figure S1 **Drug treatment modulates PIK3CA promoter but not TK promoter as revealed by luciferase activity.** 1a. A unique *PIK3CA* sensor (*PIK3CA* promoter driven *fluc2-tdt*) shows attenuation in promoter activity (luciferase activity) on treatment with cisplatin and adriamycin of transiently transfected PA1 cells (p<0.05). Treatment with paclitaxel showed decreasing trend in *PIK3CA* activity, but did not meet significance (p>0.05). 1b. Humanized renilla luciferase driven by the TK promoter (*pTK-hrl*) co-transfected in PA1 cells does not show any change in luciferase activity after drug treatment (p = ns).
(TIF)
