# Supplementary figures and images for: Non-Invasive Imaging of Phosphoinositide-3-Kinase-Catalytic-Subunit-Alpha (PIK3CA) Promoter Modulation in Small Animal Models
Source: PLoS One. 2013 Feb 5;8(2):e55971. doi: 10.1371/journal.pone.0055971 (PMC3564913; doi:10.1371/journal.pone.0055971)

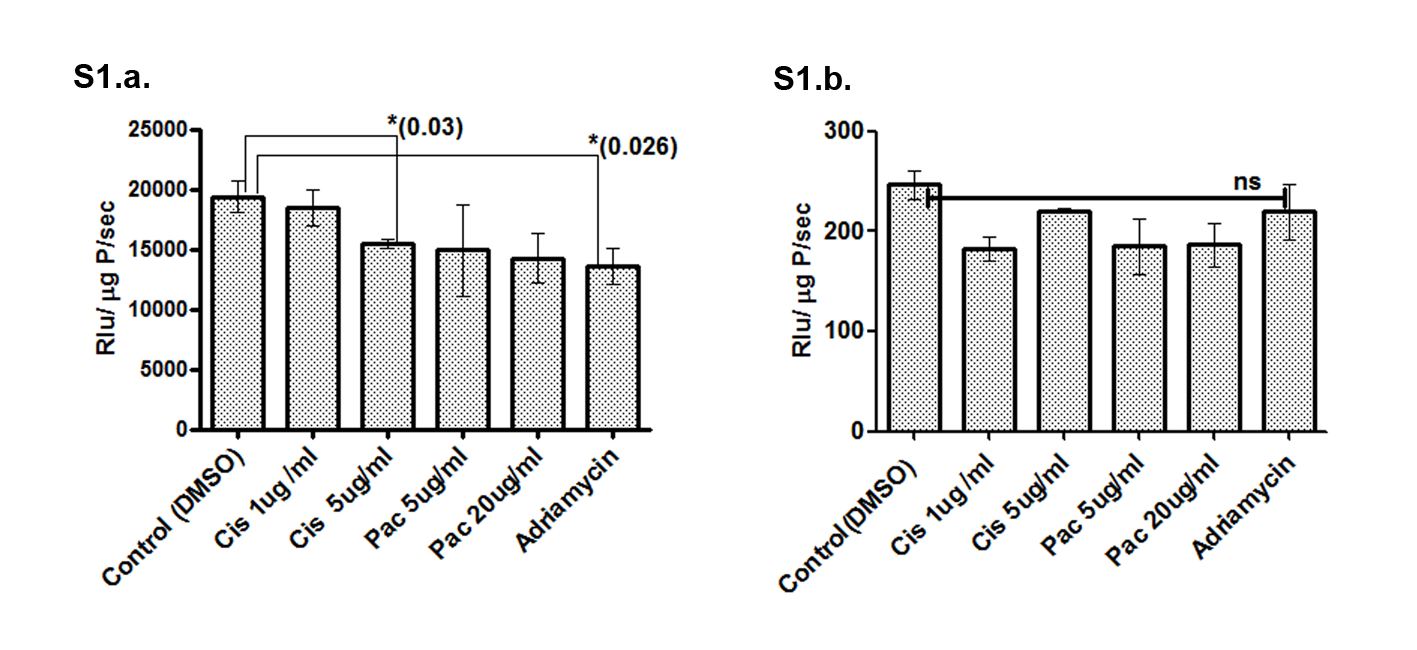

Supplement: Figure S1 — Drug treatment modulates PIK3CA promoter but not TK promoter as revealed by luciferase activity. 1a. A unique PIK3CA sensor (PIK3CA promoter driven fluc2-tdt) shows attenuation in promoter activity (luciferase activity) on treatment with cisplatin and adriamycin of transiently transfected PA1 cells (p<0.05). Treatment with paclitaxel showed decreasing trend in PIK3CA activity, but did not meet significance (p>0.05). 1b. Humanized renilla luciferase driven by the TK promoter (pTK-hrl) co-transfected in PA1 cells does not show any change in luciferase activity after drug treatment (p = ns). (TIF) [file pone.0055971.s001.tif]
